# Quid Pro Quo? A Critical Perspective on the Global Flow and Spread of Health Innovation

**DOI:** 10.34172/ijhpm.9151

**Published:** 2025-10-18

**Authors:** Russell Mannion, Ewen Speed

**Affiliations:** ^1^Health Services Management Centre, University of Birmingham, Birmingham, UK.; ^2^School of Health and Social Care, University of Essex, Colchester, UK.

**Keywords:** Health Innovation, Global Health, Decolonization, Reciprocal Innovation, Reverse Innovation, Philanthropocapitalism

## Abstract

Over recent decades, the exchange of health innovations between high-income countries (HICs) and low- and middle-income countries (LMICs) has grown significantly. Three main types of cross-border flows characterise this global health innovation ecosystem: (*i*) trickle-down innovation – where innovations originating in HICs gradually diffuse to LMICs, (*ii*) reverse innovation, where new solutions originating in LMICs are adopted and adapted in HICs, and (*iii*) reciprocal innovation – where the focus is on bidirectional exchange and learning between HICs and LMICs. Despite embracing multidirectional flows, the contemporary global health innovation ecosystem is fundamentally shaped by neocolonial power imbalances that prevent LMICs from fully benefiting. These dynamics are further intensified by recent cuts to foreign aid and the rise of philanthrocapitalism, both of which concentrate power and influence in HICs. Viewing health innovation through a neocolonial lens reveals how the current innovation ecosystem reinforces historical patterns of dependency and domination in global health.

## Mapping the Global Health Innovation Landscape

 Technological and service innovations are central to sustainable transformation in health systems worldwide. A notable trend in global health is the growing exchange of health innovations (new ideas, technological solutions, and different models of care) between high-income countries (HICs) and low- and middle-income countries (LMICs), reflecting the interconnected nature of modern health challenges.This trend represents a modern iteration of a historical pattern in which health knowledge has travelled across international borders through trade, conquest, migration, and scholarship. Early trade networks, most famously the Silk Road, were crucial conduits for the reciprocal exchange of medical knowledge, practices, and materials between distant regions and cultures. Fast-forwarding to the 21st century reveals a landscape where global healthcare is undergoing a profound transformation driven by technologies such as generative artificial intelligence (AI), cloud computing, robotics, and blockchain. Against this background, the contemporary global health innovation ecosystem has evolved into a complex network of diverse actors, including nation-states, international organisations, universities, for-profit corporations, and philanthropic foundations, all with varying mandates and interests that can lead to conflict and incompatibility in their efforts to advance global health. Given this tangled web of private, public, and charitable interests, it is genuinely difficult to distinguish between altruistic motives and market-driven profiteering. This interaction is often characterised by economic dependencies with colonial origins, leading to exploitative practices in which commercial considerations drive innovation at the expense of mutually beneficial outcomes. Such exploitative commercial practices have resulted in a net appropriation of wealth to HICs that significantly surpasses the amount of foreign aid LMICs receive.^1^ One of the most egregious examples of this is the aggressive and misleading marketing practices of formula milk companies, particularly in LMICs. By distorting or misusing scientific evidence and preying on parental anxieties, these companies boost sales at the expense of maternal and infant health by undermining the proven benefits of breastfeeding.^2^

## Trickle-Down Innovation

 The trickle-down model is the traditional and still-dominant approach where innovations are developed in HICs and then gradually trickle down to LMICs. In many ways, this unidirectional flow of innovation mirrors historical colonial power dynamics, where LMICs are cast as passive recipients of aid rather than equal partners in innovation. In some cases, multinational corporations have been accused of pressuring LMICs to address specific health issues, then advocating for solutions that benefit them financially rather than prioritising population wellbeing. Moreover, given the resource imbalances inherent in global health, LMICs’ participation in priority-setting is often minimal or tokenistic, resulting in health agendas that do not address local needs or leverage local expertise. The withdrawal of the United States from the World Health Organization (WHO)^3^ and the reduction of US overseas aid in 2025 have created a substantial gap in global health leadership and financing. This void is being increasingly filled by philanthropic foundations and other non-governmental organisations operating under a model known as philanthrocapitalism, which blends business principles with market-based strategies to philanthropic giving.^4^ Emblematic of this trend is the Bill and Melinda Gates Foundation, which uses its immense wealth and strategic network diplomacy to influence global health by shaping agendas and securing government co-investment in its priorities, creating a significant non-state influence that rivals that of some governments. While proponents see philanthropocapitalism as an effective model for delivering health technologies at lower cost and leveraging new investments, critics raise concerns that it concentrates power in the hands of an unelected elite, allowing them to shape global agendas with reduced transparency and democratic oversight.^5^ Beyond issues of governance, critics voice concerns that philanthrocapitalism favours rapid, technological “magic bullet” solutions over sustained investment in robust public health systems and addressing the root socio-economic determinants of health.^5^ A significant portion of philanthropic funding is “earmarked,” meaning that the funders, not local communities, decide how the money is spent. Furthermore, philanthrocapitalist foundations focus on leveraging additional resources from the private sector to maximise the impact of their giving, often through strategies such as public-private partnerships. The privatisation of healthcare in LMICs gives rise to concerns about whether an essential public good is being treated as an opportunity for profit-making and extractive practice. Indeed, the Bill and Melinda Gates Foundation goes as far as mandating the involvement of private sector partners as a prerequisite for their cooperation with government innovation projects, leading to the *de facto* privatisation of many essential health services in LMICs.^6^

 To understand the complex exchange relationships between HICs and LMICs in global health it is instructive to draw on the concepts of reverse innovation (innovations from LMIC benefitting HICs) and reciprocal innovation (the bi-directional exchange of knowledge and resources between LMICs and HICs).

## Reverse Innovation

 Reverse innovation is the process by which innovations that have been developed in LMICs are adopted and adapted for use in HICs.^7^ This model challenges the traditional one-way flow of ideas from HICs to LMICs and highlights how cost-effective and frugal innovative solutions born from ingenuity in resource-constrained environments can address unique challenges in wealthy nations. For example, in their analysis of global health partnerships, Syed and colleagues identified ten key areas where HICs could learn from solutions developed in resource-constrained environments, including rural health service delivery, skills substitution, and social entrepreneurship.^8^ There is a wide range of examples of reverse innovations in healthcare, including innovations in leadership, governance, and accreditation systems; health system reforms and twinning partnerships where HIC hospitals learn from LMIC counterparts.Nevertheless, it would be a mistake to assume that all innovations developed in LMICs are automatically transferable and can or should find an appropriate contextual home in HICs. Innovators from low-resource settings may encounter numerous obstacles that impede their products from being successfully adopted and scaled up in HICs. These include the need to navigate complex regulatory environments, negotiating intellectual property rights, the requirement for tailored market research to align with local needs and demands, and significant differences in culture, infrastructure, and national characteristics. Furthermore, LMIC partners who originate innovations often receive inadequate recognition or fair financial compensation for innovations that are later commercialised in HICs.^9^ Viewed through a post-colonial lens, this could be viewed as asset stripping or the uncompensated appropriation of intellectual property, perpetuating historical inequities, even while seemingly inverting the traditional flow of innovation. Indeed, the term “reverse innovation,” while presented as beneficial, could be considered oxymoronic and patronising, because it unintentionally perpetuates colonial era narratives in suggesting it is an anomaly to the normal, HIC-centric flow of innovation, with HICs the default centre of innovation.^9^ WhileSors and colleagues^10^ frame reverse innovation in terms of expertise and technology, the broader literature tends to focus much more on questions of non-material innovation than material technologies or expertise. We would argue that this misframing is a fundamental problem underpinning the reverse innovation concept, which raises the question of whether it would be better to be understood from the perspective of low-resource community-based ways of doing healthcare, being translated to HICs as a means of adapting to the increasing under-resourcing of essential services in HICs.

## Reciprocal Innovation

 Reciprocal innovation has been promoted as an alternative paradigm for global health innovation.^10^ It is premised on the assumption that health innovations, regardless of their origin, can be successfully adopted, adapted, and implemented across diverse global settings. Evolving from the concept of reverse innovation, reciprocal innovation is defined by three core characteristics: (*i*) global health partnerships rooted in the values of reciprocity, mutual learning, and equity across partner institutions in HICs and LMICs; (*ii*) a bi-directional and co-constituted approach to identifying shared health challenges across settings in long-term engagements; and (*iii*) identification of high-quality innovations from global health partnerships for demonstration, replication, and dissemination in diverse settings. Recent examples of reciprocal innovation include mental health interventions from LMICs being used in the USA and HIV/AIDS and maternal child interventions that arose from reciprocal collaborations between Kenya and the USA.The positioning of reciprocal innovation as a solution to reverse innovations’ lack of mutuality seeks to develop a better model predicated on more equitable processes of mutual exchange. For example, Sors and colleagues^10^ describe a Kenyan HIV/AIDS care system, later implemented in Indiana, USA, that featured a “comprehensive one-stop shop” HIV clinic fully integrated with other health and social services. This integrated approach aimed to improve access to care and support for people living with HIV, as well as those with other health conditions. The rapid scale-up of HIV services, including standardised treatment algorithms and documentation, is a positive outcome of the reciprocal innovation. Still, the absence of specific details on the benefits for Kenyan innovators makes it difficult to assess whether it was a truly shared innovation journey with gains on both sides.

## Decolonising Global Health: Creating a More Equitable and Sustainable Health Innovation Landscape

 The current global health innovation ecosystem, when viewed through a neocolonial lens, reveals a cycle where power is continuously consolidated in the hands of historically privileged nations who continue to set the agenda and control the flow of funding and the transfer of ideas and technology.^9^ Many global health innovation partnerships sustain an unequal donor-recipient dynamic, with HICs providing aid and dictating priorities for LMICs.^11,12^ This can result in the extraction of resources, including data and intellectual property, with LMIC partners receiving insufficient benefits or recognition for their contributions. This dynamic is being exacerbated by severe and abrupt reductions in foreign aid that are weakening state institutions and health systems in LMICs, creating a funding gap that is now being filled by private philanthropies whose agendas do not always align with the needs and preferences of local communities. While reverse and reciprocal innovation offer benefits, they are not complete solutions for building a more equitable global innovation system, as they operate within and reinforce existing power structures rather than challenging them. The core of decolonisation involves a fundamental power shift in the global matrix of power – transferring not only financial resources but also authority over decision-making, enabling actors in LMICs to pursue self-determination and shape their own futures.^9^ This includes respecting local knowledge systems and investing in strengthening institutional capacity within LMICs. Notwithstanding considerable diversity of contexts and needs among LMICs, horizontal knowledge transfer or peer-to-peer learning between LMICs may be a more promising approach than ideas imported from vastly different, high-resource environments.^13,14^ This is because it can foster the co-creation of effective solutions that are sustainable, locally relevant, and better suited to resource-constrained environments.^15,16^ Meanwhile, HICs, on their part, cannot credibly advocate for health equity abroad while neglecting it at home and therefore need to address health inequities within their own borders as a demonstration of moral consistency and authentic commitment to the principles of global health. Without first addressing the deep-seated structural inequalities rooted in neocolonialism, health innovation risks becoming another tool for exploitation rather than a vehicle for empowerment.

## Positionality

 Both authors are senior white male academics at academic institutions in the UK. We sit on the boards of several international health policy journals and are committed to doing work that contributes to a more sustainable and equitable world. We recognise that our privileged positions and power have facilitated access to research opportunities and networks that are denied to many others. We are aware of the potential performativity in writing positionality statements such as this but offer it in good faith as part of our commitment to transparency and reflexive practice.

## Disclosure of artificial intelligence (AI) use

 Not applicable.

## Ethical issues

 Not applicable.

## Conflicts of interest

 Authors declare that they have no conflicts of interest.
